# Evaluation of the Anticancer Activities of Novel Transition Metal Complexes with Berenil and Nitroimidazole

**DOI:** 10.3390/molecules25122860

**Published:** 2020-06-21

**Authors:** Robert Czarnomysy, Dominika Radomska, Anna Muszyńska, Justyna Magdalena Hermanowicz, Izabela Prokop, Anna Bielawska, Krzysztof Bielawski

**Affiliations:** 1 Department of Synthesis and Technology of Drugs, Medical University of Bialystok, Kilinskiego 1, 15-089 Bialystok, Poland; dominika.radomska@umb.edu.pl (D.R.); anna.muszynska@umb.edu.pl (A.M.); krzysztof.bielawski@umb.edu.pl (K.B.); 2 Department of Pharmacodynamics, Medical University of Bialystok, Mickiewicza 2c, 15-222 Bialystok, Poland; justyna.hermanowicz@umb.edu.pl; 3 Department of Medicinal Chemistry, Medical University of Bialystok, Mickiewicza 2c, 15-222 Bialystok, Poland; izabela.prokop@umb.edu.pl; 4 Department of Biotechnology, Medical University of Bialystok, Kilinskiego 1, 15-089 Bialystok, Poland; anna.bielawska@umb.edu.pl

**Keywords:** anticancer compounds, multi-factorial compounds, transition metal complexes, nitroimidazole, breast cancer

## Abstract

Novel transition metal complexes (Au, Pd, Pt) with berenil and 2-(1-methyl-5-nitroimidazol-2-yl)ethanol were obtained through two-step synthesis. The cytotoxicity assay against MCF-7 and MDA-MB-231 breast cancer cells revealed that novel platinum and palladium complexes cause a reduction on the viability of MCF-7 and MDA-MB-231 breast cancer cells to a greater extent than cisplatin. The complexes showed lower cytotoxicity on normal MCF-10A human breast epithelial cells than on tumor cells. Furthermore, we observed that these complexes selectively concentrate in tumor cell mitochondria due to the characteristic for these cells increased membrane potential that may explain their increased proapoptotic activity. The activity of the synthesized compounds against topoisomerase type IIα and their increased impact on DNA defragmentation also were documented. The novel complexes also induced autophagosome changes and inhibited tumor growth in xenograft models (established using breast cancer cells).

## 1. Introduction

Despite the development of thousands of anticancer structures, cancer is still a major cause of death worldwide [1]. The main problem in the treatment of cancer is drug resistance from long-term treatment and the adverse side effects of the drugs along with non-specific toxicity [1,2]. In addition, the reported new anticancer compounds usually suffer from low stability under physiological conditions, interfering with delivery to the tumor tissues and often resulting in toxic side effects [3]. Therefore, there is a constant search for new compounds with potential anticancer effects that at the same time will be lack from these disadvantages.

Metal complexes offer unique characteristics and exceptional versatility, with the ability to alter their pharmacology through facile modifications of geometry and coordination number. This has prompted the search for metal-based complexes with distinctly different structural motifs and non-covalent modes of binding with a primary aim of circumventing current clinical limitations [4]. Another advantage of metal complex therapy is their ability to mimic low-toxicity natural compounds [5]. Metal centers also serve as unique 3-dimentional structural scaffolds that can be adjusted to fit into the base stacks and grooves of targeted DNA sequences [6].

In recent years, there have been more and more scientific reports about imidazole and the related azole moiety. The imidazole ring is an important five-membered aromatic heterocycle widely present in natural products and synthetic molecules. Due to the two nitrogen atoms present in the structure, the imidazole ring has the ability to form hydrogen bonds and thus is favorable to improve the water solubility of target compounds. For this reason, the imidazole ring has been considered a valuable structural fragment and frequently introduced into other bioactive skeletons. Imidazoles could interfere with DNA synthesis via weak interactions such as coordination bonds, hydrogen bonds, and π–π stacking and then halt cell growth and division. Furthermore, these chemical structures could easily bind to protein molecules compared with other heterocyclic rings inhibiting the synthesis of essential cell membrane components [7,8]. From the point of view of the medicinal chemist, imidazole derivatives are very important ligands in bioinorganic chemistry as they can provide bidentate N-donor sites for the synthesis of new transition metal analogues [9]. They have great potentiality to overcome the diverse drawbacks of currently available clinical drugs and to be developed as anticancer drugs.

Continuous effort has been directed toward developing new imidazole-based anticancer agents targeting various enzymes or receptors such as microtubule, topoisomerases, cytochrome P450 enzyme, transforming growth factor-β (TGF-β) and DNA [10,11]. We therefore designed a series of novel transition metal complexes with berenil and nitroimidazole. Imidazole moiety allowed us to increase molecular stability that may improve the physicochemical property and anchor an electron-rich aromatic ring on the N-moiety that helps to enhance antitumor activity.

## 2. Results

### 2.1. Chemistry Section

**Figure 1:**
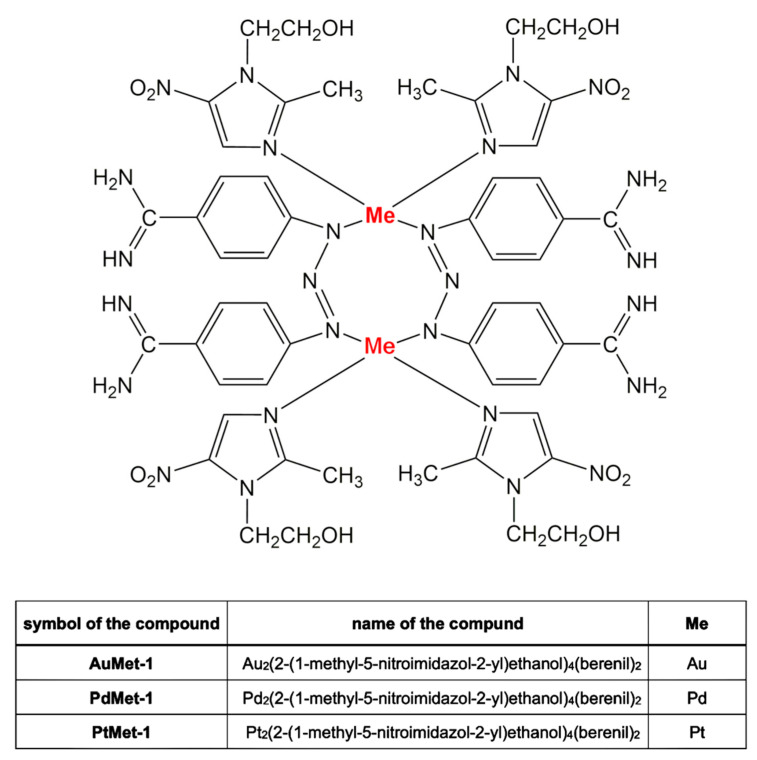
Structures of novel transition metal complexes (Au, Pd, Pt) with berenil and 2-(1-methyl-5-nitroimidazol-2-yl)ethanol.

New transition metal complexes (Au, Pd, Pt, Figure 1) with berenil and 2-(1-methyl-5-nitroimidazol-2-yl)ethanol were obtained through two-step synthesis. In the first stage of the synthesis, inorganic metal salts of gold, palladium and platinum were treated with 2-(1-methyl-5-nitroimidazol-2-yl) ethanol to give stable intermediates. After isolation and purification, the obtained intermediates were used for the second stage. For this purpose, the previously obtained compounds were treated with an aqueous solution of AgNO_3_ followed by berenil in a 10% NaCl. Since it is known that berenil, has functional and triazene groups, it may bind to metals as linear monodentate, chelating bidentate, and in bridging bidentate modes, we performed IR, ^1^H-, and ^13^C-NMR spectra of the synthesized compounds to determine the mode of metal-berenil bonding. The spectra show that binding of the metal to the amidino-group of the ligand does not occur since the frequencies attributed to amidino-moiety, v(CN) = 1686–1687 cm^−1^ (for free berenil 1668 cm^−1^) as well as the signal in ^13^C-NMR of the carbon from the amidine group remain unaltered relative to the free ligand, 165.2 ppm. The presence of two broad singlets between 9.24–9.48 ppm attributed to the protonated residue in ^1^H-NMR indicate, moreover, the absence of this type of metal bonding. The presence of three bands at 1606–1608, 1256–1259, 1173–1175 cm^−1^ in the IR spectrum shows, on the other hand, that the triazene group coordinates in linear or bridging modes. The IH- and ^13^C-NMR spectra further support this mode of binding since the signal corresponding to the AA′BB′ system of the nearest protons of the triazene group are now broadened and distorted. Thus, the spectra and the micronalytical data, together with the spectrum of AuMet-1, PdMet-1, PtMet-1, suggest that the compounds are dimers, where the metal binds to the nitrogen atoms of two triazene groups in bridging mode.

### 2.2. Biological Activity of Novel Series of Transition Metal Complexes with Nitroimidazole and Berenil (AuMet-1, PdMet-1, PtMet-1)

**Figure 2:**
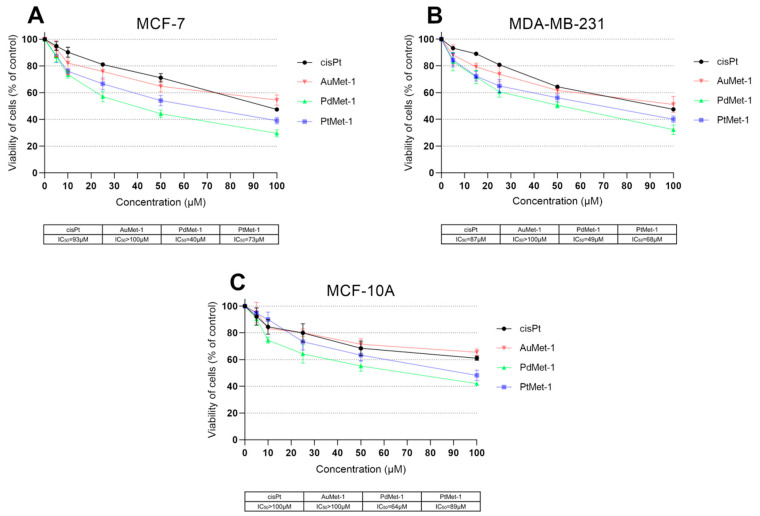
Viability of MCF-7 (**A**) and MDA-MB-231 (**B**) breast cancer cells and normal human breast epithelial cell MCF-10A (**C**) treated for 24 h with different concentrations of the tested compounds: AuMet-1, PdMet-1, PtMet-1 and cisplatin. Mean values ±SD from 3 independent experiments (n = 3) done in duplicate are presented.

In the first stage, the effect of a series of transition metal (Au, Pd, Pt) compounds with nitroimidazole and berenil moiety on the viability of breast cancer cells MCF-7, MDA-MB-231 and non-tumorigenic epithelial breast cell line MCF-10A was analyzed using an MTT assay (Figure 2). The IC_50_ values for the reference drug (cisplatin) were 93 µM in MCF-7 cells, 87 µM in MDA-MB-231 cells and >100 µM in MCF-10A cells after 24 h of incubation. It was shown that the AuMet-1 compound was less cytotoxic than cisplatin in all cell lines (IC_50_ > 100 µM). In the case of the PdMet-1 and PtMet-1 compounds in MCF-7 cells, the IC_50_ values were 40 µM for PdMet-1 and 73 µM for PtMet-1, while in MDA-MB-231 cells 49 µM for PdMet-1 and 68 µM for PtMet-1 after 24 h of incubation. In MCF-10A cells, the obtained IC_50_ values were 64 µM for PdMet-1 and 89 µM for PtMet-1. The compound that exhibited the highest cytotoxic properties was PdMet-1.

### 2.3. Novel Series of Transition Metal Compounds with Nitroimidazole and Berenil Moiety (AuMet-1, PdMet-1, PtMet-1) Induce Apoptosis by Decreasing Mitochondrial Membrane Potential

**Figure 3:**
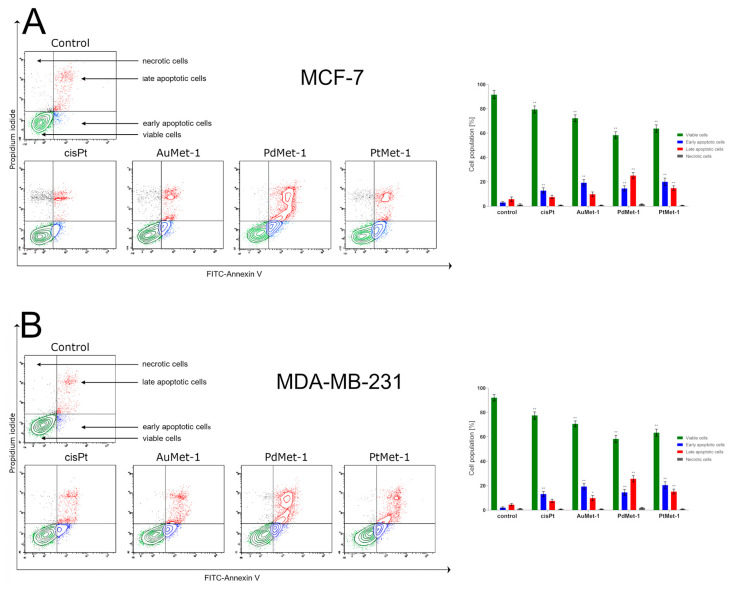
Flow cytometry analysis of MCF-7 (**A**) and MDA-MB-231 (**B**) breast cancer cells after 24-h incubation with AuMet-1, PdMet-1, PtMet-1 and cisplatin (50 μM) and subsequent staining with Annexin V and propidium iodide. Mean percentage values from 3 independent experiments (n = 3) done in duplicate are presented. *p < 0.05 vs. control group, ** *p* < 0.01 vs. control group.

Dual annexin V and iodium propidium staining with flow cytometry were used to assess the apoptosis status of breast cancer MCF-7 and MDA-MB-231 cells after 24 h of treatment with a novel series of tested compounds (AuMet-1, PdMet-1, PtMet-1) and cisplatin (Figure 3). Annexin V served to identify cells in the early stages of apoptosis, because it detects the externalized phosphatidylserin (PS) on the cell membrane (characteristic change during apoptosis), whereas iodium propidium (PI) identified late apoptotic and dead cells. PI stains cells with a disrupted cell membrane. Live cells don’t bind to annexin V and PI (annexin-V^−^/PI^−^), early apoptotic cells bind to annexin V but not PI (annexin-V^+^/PI^−^), late apoptotic cells bind to both molecules (annexin-V^+^/PI^+^), and death cells only bind to PI (annexin-V^−^/PI^+^). 

Following 24 h of incubation with the tested compounds (concentration of 50 µM), we observed that all of them significantly induced apoptosis in MCF-7 and MDA-MB-231 cells compared with the control. In the control cells, there were 8.9 ± 2.4% apoptotic cells* and 1.3 ± 0.7% necrotic cells in the population of MCF-7 cells and 6.4 ± 1.6% apoptotic cells* and 1 ± 0.3% necrotic cells in the MDA-MB-231 cells.

The strongest proapoptopic properties on both cell lines after 24 h of incubation were exhibited by PdMet-1, where we observed 58.4 ± 2.8% viable cells and 39.7 ± 4.5% apoptotic cells* in MCF-7 cells and 58.3 ± 2.9% viable cells and 40.2 ± 4.2% apoptotic cells* in MDA-MB-231 cells. The weakest proapoptopic properties in both cell populations were exhibited by AuMet-1, where we observed 72.2 ± 2.9 (MCF-7) and 70.6 ± 2.4% (MDA-MB-231) viable cells; 29.0 ± 4.5 (MCF-7) and 29.1 ± 4.7% (MDA-MB-231) apoptotic cells*, respectively. The PtMet-1 compound also exhibited proapoptotic potential in both breast cancers (similar to PdMet-1, however the proapoptotic activity was slightly lower): MCF-7 63.8 ± 3.0% viable cells and 34.9 ± 1.4% apoptotic cells*; MDA-MB-231 63.4 ± 2.9% viable cells and 35.7 ± 0.7% apoptotic cells (the percent of cells with early and late apoptosis). At the same time, it’s worth emphasizing that in the case of cisplatin, the percentage of apoptopic cells* was only 20.4 ± 0.8% (MCF-7) and 20.6 ± 3.6% (MDA-MB-231). Based on the obtained results, we concluded that the cytotoxic activity of the novel series of transition metal (AuMet-1, PdMet-1, PtMet-1) compounds with nitroimidazole and berenil moiety against both breast cancer cells may dependent on the induction of programmed cell death.

As one of the earliest changes in the apoptotic process, a decrease in the mitochondrial membrane potential (MMP) is observed. Apoptosis, which proceeds through the mitochondrial pathway, shows an increase in the permeability of the internal and external mitochondrial membrane, which is associated with changes in the transmembrane mitochondrial potential (ΔΨm) [12]. The change of ΔΨm was determined using lipophilic fluorochrome JC-1 and flow cytometry analysis. JC-1 occurs in two forms, monomers and aggregates, which emit different fluorescence color. Fluorochrome emits green fluorescence in healthy cells (monomers) and red fluorescence in cells with disturbed mitochondrial potential (aggregates).

**Figure 4:**
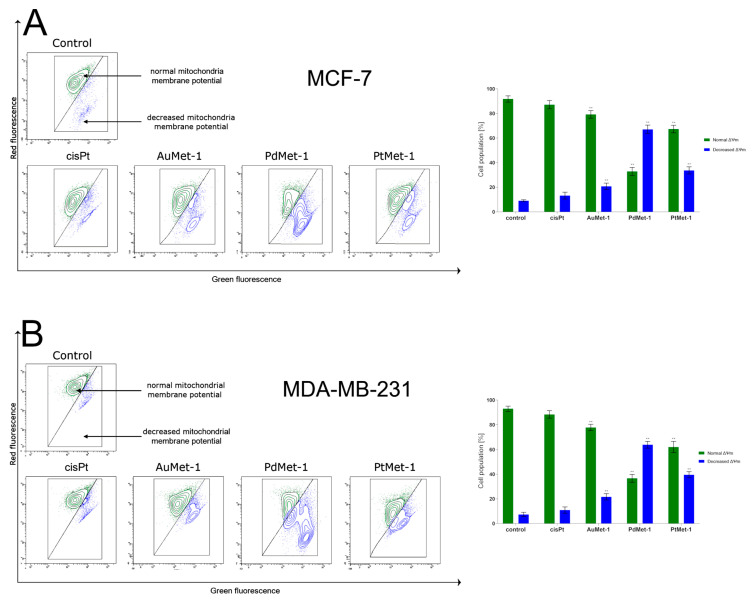
Fluorescence of MCF-7 (**A**) and MDA-MB-231 (**B**) breast cancer cells treated for 24 h with AuMet-1, PdMet-1, PtMet-1 and cisplatin (50 μM) incubated with mitochondrial membrane potential probe JC-1. Mean percentage values from three independent experiments (n = 3) done in duplicate are presented. * *p* < 0.05 vs. control group, ** *p* < 0.01 vs. control group.

As shown in Figure 4, a 24-h incubation with the tested compounds (concentration of 50 µM) caused an increase in the proportion of breast cancer cells MCF-7 and MDA-MB-231 with depolarized mitochondria. In the control group (untreated cells), the MMP decrease was 9.0 ± 0.8% in MCF-7 and 7.3 ± 1.7% in MDA-MD-231 cells, respectively. The highest MMP decrease was observed in PdMet-1 and it was 67.1 ± 3.5% in MCF-7 cells and 63.8 ± 2.7% in MDA-MB-231 cells. The MMP decrease induced by the remaining compounds was higher than the reference compound—cisplatin (MMP decrease: 13.2 ± 2.8% in MCF-7 and 10.9 ± 2.5% in MDA-MB-231). It was proved that the tested compounds had a greater effect on programmed cell death measured by annexin V binding and an MMP decrease than cisplatin. The mitochondrial membrane potential results are consistent with those obtained in the annexin V/iodium propidium test and show that programmed cell death induced by the novel series of transition metal (AuMet-1, PdMet-1, PtMet-1) compounds may go through the mitochondrial pathway.

### 2.4. AuMet-1, PdMet-1 and PtMet-1 Induce Autophagy

**Figure 5:**
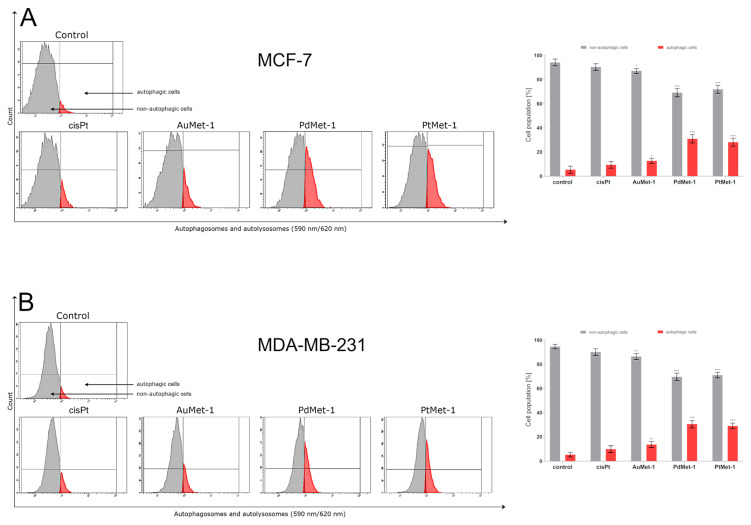
Autophagy induction in MCF-7 (**A**) and MDA-MB-231 (**B**) breast cancer cells measured by flow cytometry using Autophagy Probe (right-red histogram) compared to negative control cells (left- gray histogram) after 24-h incubation with AuMet-1, PdMet-1, PtMet-1 and cisplatin (50 µM). Mean percentage values from 3 independent experiments (n = 3) done in duplicate are presented. * *p* < 0.05 vs. control group, ** *p* < 0.01 vs. control group, *** *p* < 0.001 vs. control group.

Autophagy is one of the most important processes to maintain internal cell homeostasis. In healthy cells, it occurs at a low level because its role is to prevent the accumulation of damaged cell organelles, but its level increases during starvation or stress [13]. Autophagy Probe, Red staining with flow cytometry was used to assess the state of autophagy of breast cancer cells MCF-7 and MDA-MB-231 after 24 h of exposure to the tested compounds in a concentration of 50 µM (Figure 5). Autophagic Probe, Red identifies autophages and autolysosomes by introducing molecules into the lipid membranes of these structures and then bright fluorescence.

After 24 h of incubation, we observed that all the tested compounds induced autophagy in MCF-7 and MDA-MB-231 cells with a different intensity compared to the control (untreated cells). There was 94.2 ± 2.8% of non-autophagic cells and 5.3 ± 3.0% of autophagic cells in the control population of MCF-7 cells and 94.6 ± 1.9% of non-autophagic cells and 5.3 ± 1.9% of autophagic cells in the MDA-MB-231 cells. The highest activation of autophagy on both cell lines after 24-h incubation was exhibited by PdMet-1, where we observed 30.9 ± 3.5% of autophagic cells in MCF-7 and 30.4 ± 3.1% in MDA-MB-231 cells. An insignificant intensity of autophagy was observed in cisplatin (9.4 ± 2.8% of autophagic cells in MCF-7 and 9.9 ± 2.4% in MDA-MB-231). In the case of the AuMet-1 compound, the activation of autophagy was higher than in cisplatin: 12.7 ± 2.0% of autophagic cells in MCF-7 and 13.6 ± 2.4% in MDA-MB-231; but more than two-fold lower than in PdMet-1 and PtMet-1: 30.9 ± 3.5% and 28.1 ± 3.3% in MCF-7, 30.4 ± 3.1% and 29.0 ± 2.3% in MDA-MB-231, respectively.

Autophagy allows to maintain homeostasis in the cell, thus avoiding its death. If cellular stress is not overcome, which leads to a homeostasis disorder, the same signaling ultimately leads to the activation of programmed cell death [14]. Thanks to this knowledge we can conclude that compounds PdMet-1 and PtMet-1 cause large stress in cells, which leads to the disruption of their homeostasis and directing the cells to apoptosis, which is proved in the annexin-V/PI assay.

### 2.5. AuMet-1, PdMet-1, and PtMet-1 Increase Topoisomerase IIα Activity

**Figure 6:**
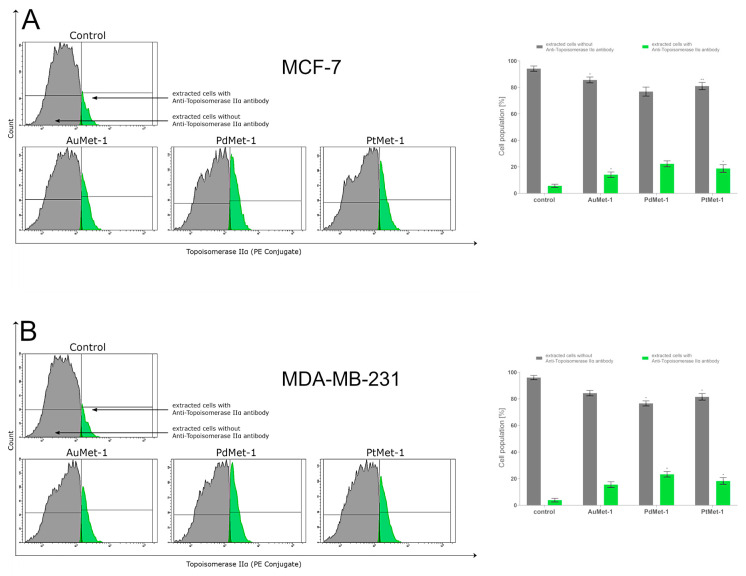
Anti-topoisomerase IIα Antibody flow cytometric analysis of MCF-7 (**A**) and MDA-MB-231 breast cancer cells (**B**) (right histogram- green color) compared to a negative control cell (left histogram- gray color) after 24 h of incubation with AuMet-1, PdMet-1, PtMet-1 and cisplatin (50 µM). Mean percentage values from 3 independent experiments (n = 3) done in duplicate are presented. * *p* < 0.05 vs. control group, ** *p* < 0.01 vs. control group.

Topoisomerase IIα is an enzyme responsible for the correct process of replication, transcription and recombination. It takes part, among others, in chromosome condensation or separation of chromatids. Scientific research has proved that the overexpression of this enzyme generates double-strand breaks of DNA (DSB) induced by topoisomerase IIα, directing cells to the apoptosis pathway [15]. The activity of topoisomerase IIα was measured using anti-topoisomerase IIα antibody conjugated with phycoeritin and flow cytometer analysis of breast cancer cells MCF-7 and MDA-MB-231 after 24 h of treatment with AuMet-1, PdMet-1, and PtMet-1 (Figure 6). All of them in a concentration of 50 µM. The antibody was binding with topoisomerase IIα in cancer cells and brightly fluoresced. It was observed that all the tested compounds induced topoisomerase IIα activity in MCF-7 and MDA-MB-231 cells with a different intensity compared to the control (untreated cells). In the control group, there were 94.2 ± 2.0% in MCF-7 and 95.3 ± 2.8% in MDA-MB-231 cells without antibody and 5.7 ± 1.2% in MCF-7 and 4.5 ± 2.5% in MDA-MB-231 cells with anti-topoisomerase IIα antibody. The highest activity of topoisomerase IIα on both cell lines after 24-h incubation was exhibited by PdMet-1, where we observed 77.2 ± 2.9% of cells without antibody and 22.7 ± 2.2% with antibody in MCF-7 and 76.5 ± 2.0% of cells without antibody and 23.3% ± 2.1 with antibody in MDA-MB-231. The compound exhibiting the lowest activation of topoisomerase IIα was AuMet-1; after 24-h incubation, there was 85.6 ± 2.2% of cells without antibody and 14.0 ± 2.1% of cells with anti-topoisomerase IIα antibody in MCF-7 and 84.3 ± 2.1% and 15.5 ± 2.1% in MDA-MB-231. The PtMet-1 compound induced topoisomerase IIα activity insignificantly more than the AuMet-1 compound and was less effective than PdMet-1: PtMet-1: 81.0 ± 2.8% in MCF-7 and 81.5 ± 2.5% in MDA-MB-231 cells without antibody and 18.8 ± 2.9% in MCF-7 and 18.3 ± 2.5% in MDA-MB-231 cells with antibody. These results are consistent with those obtained in the annexin V/iodium propidium and MMP tests and show that programmed cell death induced by PdMet-1 may also be related to increased topoisomerase IIα activity.

### 2.6. AuMet-1, PdMet-1 and PtMet-1 Promote DNA Fragmentation

**Figure 7:**
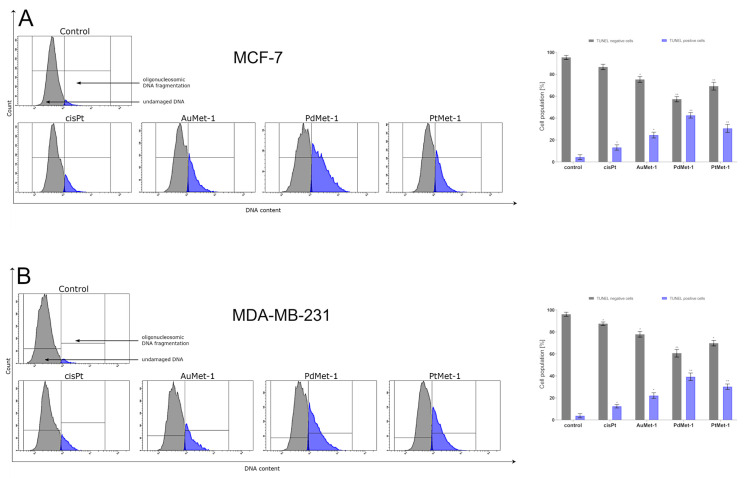
Flow cytometric analysis of DNA fragmentation of MCF-7 (**A**) and MDA-MB-231 (**B**) breast cancer cells after 24 h of incubation with AuMet-1, PdMet-1, PtMet-1 and cisplatin (50 μM) using TUNEL assay. Histograms present TUNEL negative and TUNEL positive cells. Mean percentage values from 3 independent experiments (n = 3) done in duplicate are presented. * *p* < 0.05 vs. control group, ** *p* < 0.01 vs. control group.

DNA damage can be the result of external factors such as ionizing and ultraviolet radiation, cytotoxic or internal factors, which are: reactive oxygen species and DNA polymerase errors during replication [16]. The above factors may lead to single- and double-strand DNA breaks, which are identified by the TUNEL assay. This test uses the incorporation of FITC-labelled nucleotides by using an exogenous terminal deoxynucleotide transferase (TdT). The FITC-labelled nucleotides incorporated into DNA emit green fluorescence. As shown in Figure 7, 24-h incubation with the tested compounds (concentration of 50 µM) caused an increase in the proportion of breast cancer cells MCF-7 and MDA-MB-231 with DNA fragmentation.

In the untreated cells (control), the TUNEL positive cells was 4.4 ± 2.1% in MCF-7 and 3.8 ± 1.9% in MDA-MB-231, respectively. In case of PdMet-1 we detected the highest percentage of TUNEL positive cells − it was 42.5 ± 2.5% in MCF-7 and 39.2 ± 3.5% in MDA-MB-231, respectively. AuMet-1 and PtMet-1 showed a lower percentage of TUNEL positive cells compared with PdMet-1 in both cell lines: 24.5 ± 2.7% (MCF-7) and 22.1 ± 2.5% (MDA-MB-231), 30.6 ± 3.6% (MCF-7) and 30.2 ± 2.5% (MDA-MB-231), respectively. We found that all the tested compounds caused DNA strand breaks to a greater extent than the reference compound (cisplatin).

### 2.7. PdMet-1 and PtMet-1 Inhibit Tumor Development in Zebrafish Xenografts

Before transplantation, MCF-7 and MDA-MB-231 were labelled with CM-Dil (chloromethylbenzamino derivatives of 1,1′-dioctadecyl-3,3,3′,3′-tetramethylindocarbocyanine perchlorate), prepared as recommended by the manufacturer (Thermo Fisher Scientific Inc., Waltham, MA, USA). Cells in the final concentration of 1 × 10^7^ cells/mL were implanted into the yolk sac of wild type (WT) zebrafish 48 hpf embryos. Three days after cell injection, solid tumors were established in 100% of MCF-7 and MDA-MB-231 xenografts (n = 20).

**Figure 8:**
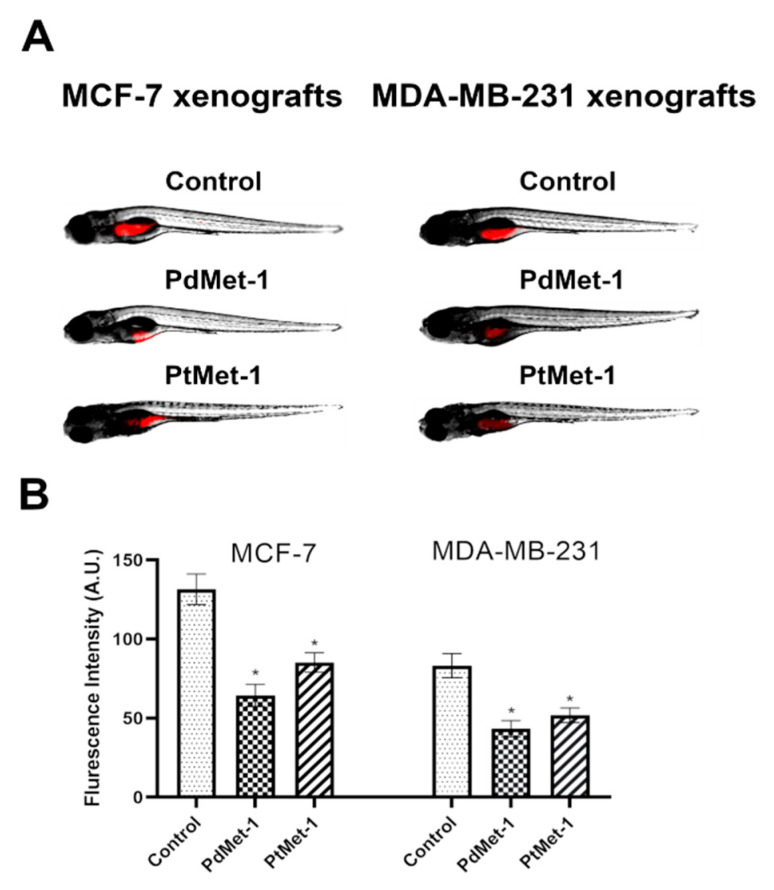
Site-specific injection (yolk sac) of transfected (red) breast cancer cells (MCF-7 and MDA-MB-231) into 48 hpf zebrafish embryos and imaging analysis of tumor growth after 48 h of incubation with PdMet-1 and PtMet-1 (100 µM) (**A**). Quantification of total mCherry fluorescence by breast cancer cells 3 days after injection (**B**) n = 4, * *p* < 0.05.

The fish were incubated with PdMet-1 and PtMet-1 for 48 h. The control group received a solvent for the tested compounds (1% DMSO). In both the MCF-7 and the MDA-MB-231 xenografts, we observed a significant reduction in tumor development in the PdMet-1 and PtMet-1 treated group compared with the control as reflected by the drop in fluorescence intensity (64.4 ± 7.1 and 85.2 ± 6.2 vs. 131.5 ± 10.86; and 51.8 ± 4.6 and ± vs. 83.2 ± 7.6, respectively; *p < 0.05) (Figure 8). PdMet-1 showed a markedly higher anticancer effect than PtMet-1.

## 3. Discussion

Despite the fact that currently cisplatin has a leading, well-established position among drugs used in the treatment of cancer, its use is limited by the toxic side effects. Therefore much research aims at the synthesis of new complexes directed at different targets. The presence of a less labile ligand in the structure of new generation platinum drugs may contribute to the lower toxicity of these compounds, especially neuro- and nephron- toxicity, while maintaining the same, and in some cases greater, antitumor activity [17,18]. The compounds synthesized by our team seem to confirm this thesis.

In our research, we conducted studies on the anti-tumor activity of three transition metal (Au, Pd, Pt) complexes with nitroimidazole ligand. The cytotoxicity assay against breast cancer cells MCF-7 and MDA-MB-231 revealed that the novel compounds were more effective than the hitherto used agent, cisplatin. At the same time, what is worth emphasizing is that the tested compounds showed lower cytotoxicity on normal human breast epithelial cells MCF-10A than on tumor cells. Moreover, the fact that they have high cytotoxic activity at lower levels than cisplatin is important. This may suggest the high activity of the nitroimidazole ligand present in the complex. Our research confirms the observation of earlier research teams that suggested that nitroimidazoles may have antiproliferation activity in an appropriate chemical system [19]. In addition, we observed that this activity varies depending on the current central atom present in the structure. The cytotoxic activity of these compounds with a different central atom increased in the following order: PdMet-1 > PtMet-1 > AuMet-1.

The anticancer action of PdMet-1 and PtMet-1 was also investigated in a zebrafish embryo xenograft model. The xenograft experiment using MCF-7 and MDA-MB-231 human breast cancer cells grafted into the zebrafish yolk showed the significant antitumor effect of these compounds compared with the control group. According to the research carried out by Rawera et al. [20], the higher anti-tumor activity of compounds with these central atoms may be due to their greater intracellular penetration capacity. These authors noted that platinum(II) complexes with imidazole ligands have the ability to decrease the side effects and regulate the hydrophilicity/hydrophobicity ratio, which makes them easy to penetrate into biofilms, thus leading to a better curative effect.

The search for new drugs based on cisplatin is mainly associated with the mechanisms of cellular resistance to this medicine. The resistance of cancer cells to platinum compounds is a process that probably consists of many factors. First of all, the mechanisms of intracellular resistance, which include: increased activity of DNA repair processes and increased tolerance to DNA deformations, should be considered [21]. The key enzymes involved in DNA repair are topoisomerases. They perform their function by introducing transient protein-bridged DNA breaks on one (topoisomerase I) or both DNA strands (topoisomerase II). They have been recognized as a valuable and particularly intriguing target for chemotherapeutics because of their critical roles in cell progression, apoptosis, transcription, and other cellular regulation. Topoisomerases targeting agents could not only stabilize the cleavable complex formed between the enzyme and DNA, but also control the replication and transcription of DNA in malignant tumor cells [22].

Our study using topoisomerase IIα antibody showed that the tested compounds induced excessive expression of this enzyme. The imidazole ring may be responsible for this activity. Its unique structural feature with desirable electron rich characteristic is beneficial, because it enables to readily bind with a variety of enzymes and receptors in biological systems through diverse weak interactions, thereby exhibiting broad bioactivities [22]. Our thesis seems to be confirmed by the research of Shinohar at al., who reported that imidazole polyamides could competitively inhibit nuclear factor-Y (NF-Y) binding to the ICB2 site in the topoisomerase IIα promoter region and cause the upregulation of this enzyme [23]. Topoisomerase IIα increased activity could potentially lead to DNA fragmentation resulting in cell death by apoptosis [24]. The induction of apoptosis as result of DNA damage in precancerous lesions can remove potentially harmful cells, thereby blocking tumor growth [25]. We observed inhibited tumor growth as well as increased induction of apoptosis and DNA defragmentation under the influence of the tested compounds in our research. While examining the effect of the tested compounds on the process of apoptosis, we noticed significant accumulation of apoptotic breast cancer cells with externalized phosphatidylserine after incubation with the tested compounds. To explore the cellular mechanism underlying the synthetized compounds inducing apoptosis, we examined the alterations of the mitochondrial transmembrane potential (MMP) by using flow cytometry analysis. We found that all of the new derivatives increased the number of cells with decreased levels of MMP compared with the control cells in both lines. In cancer, the apoptotic pathway is typically inhibited through a wide variety of means including overexpression of antiapoptotic proteins and underexpression of proapoptotic proteins. Many of these changes cause intrinsic resistance to the most common anticancer therapy, chemotherapy. Therefore compounds that exhibit anticancer activity through activating the apoptotic pathway are the promising new anticancer therapies [26].

Researchers have already confirmed that novel compounds with potential anticancer activity based on cisplatin exert antitumor effects by inducing autophagy [27]. Moreover some effective anticancer agents, such as rapamycin and nilotinib, also repress cancer cell growth through the induction of autophagy [28,29]. Autophagy exhibits either a protumorigenic or antitumorigenic function, depending on the cell type, developmental stage of cancer, and stimulator [14]. Previously, Chen K. et al. demonstrated that stimulation of autophagy could be a potential strategy for the treatment of breast cancer [30]. In this study, we showed that autophagy induced by the tested compounds had an anticancer effect against breast cancer. We found that the induction of autophagy was involved in the cytotoxicity of a novel series of transition metal compounds. Considering the cross link between autophagy and apoptosis, we assume that the autophagy pathway could be involved in cytotoxicity and ultimately tumor reduction by the tested compounds.

## 4. Materials and Methods 

### 4.1. Materials

Potassium gold(III) chloride, potassium tetrachloropalladate(II), potassium tetrachloroplatinate(II), potassium iodide, acetone, 2-(1-methyl-5-nitroimidazol-2-yl)ethanol, nitric acid(V) silver(I) salt, methanol, diethyl ether, cisplatin, 3-(4,5-dimethylthiazol-2-yl)-2,5-diphenyltetrazolium bromide (MTT), formaldehyde, DMSO were purchased from Sigma Chemical Co. (St. Louis, MO, USA). Stock cultures of human breast cancer cell (MCF-7 and MDA-MB-231) and normal human breast epithelial cell (MCF-10A) were purchased from the American Type Culture Collection (ATCC, Manassas, VA, USA). Dulbecco’s minimal essential medium (DMEM), fetal bovine serum (FBS), PBS used in a cell culture, glutamine, penicillin, and streptomycin were obtained from Gibco (San Diego, CA, USA). MEGM Mammary Epithelial Cell Growth Medium BulletKit was purchased from Lonza Bioscience (Basel, Switzerland). FITC Annexin V Apoptosis Detection Kit II, JC-1 MitoScreen Kit and APO-Direct Kit were from BD Pharmigen (San Diego, CA, USA), Autophagy Assay, Red (ImmunoChemistry Technologies, Bloomington, MN, USA), Topoisomerase IIα- PE Conjugate (Cell Signaling Technology, Beverly, MA, USA). Zebrafish were obtained from the Centre for Experimental Medicine of the Medical University of Lublin Bialystok, Poland.

### 4.2. Physical Measurements

The structure of a synthesized compound was confirmed by ^1^H-NMR (200 MHz) and ^13^C-NMR (50 MHz) spectra recorded on an AC 200F instrument (Bruker Hamburg, Germany) in deuterated dimethylsulfoxide (DMSO-*d*_6_). Chemical shifts were expressed as a δ value (ppm). The multiplicity of resonance peaks was indicated as a singlet (*s*), doublet (*d*), triplet (*t*), quartet (*q*), and multiplet (*m*). Infrared spectra were recorded on a Spectrum 100 FT-IR spectrometer (Perkin-Elmer, Houston, TX, USA) as KBr pellets (4000–450 cm^−1^). Mass spectra were recorded using a Mariner mass spectrometer (company, Waltham, MA, USA). Melting points were determined on a 535 melting-point apparatus (Buchi, Essen, Germany), and are uncorrected. Elemental analysis of C, H, and N was performed on a Perkin-Elmer 240 analyzer and satisfactory results within ± 0.4% of the calculated values were obtained.

### 4.3. Chemistry

#### 4.3.1. Preparation of [Au_2_(2-(1-methyl-5-nitroimidazol-2-yl)ethanol)_4_(berenil)_2_]·4HCl·2H_2_O (**AuMet-1**)

KAuCl_4_ (1.00 mmol) was dissolved in deionized water (30 mL). Then, 2-(1-methyl-5-nitroimidazol-2-yl)ethanol (2.00 mmol) was added to the reaction mixture and gradual dissolution of the substrate was observed. Stirring was continued for 24 h and the newly formed precipitate was then collected by filtration. This compound (Au(2-(1-methyl-5-nitroimidazol-2-yl)ethanol)_2_Cl_2_) was filtered, then washed with 20 mL deionized water and dried under vacuum. Au(2-(1-methyl-5-nitroimidazol-2-yl)ethanol)_2_Cl_2_ (0.62 mmol) was suspended in an aqueous solution of silver nitrate (AgNO_3_, 5 mL, 1.25 mmol). The reaction mixture was stirred for 24 h at room temperature in the dark. The AgCl precipitate was filtered off. Berenil (0.62 mmol) and a solution of 10% NaCl (5 mL) were added to the filtrate and stirred for 24 h until a precipitate of the final gold complex formed. Afterwards, the product was filtered off and washed with a small amount of diluted HCl, deionized water, and ethyl ether, and dried under vacuum. Yield: 27.2% (151.32 mg); olive powder; mp 166–168 °C; ^1^H-NMR (DMSO-*d*_6_) δ (ppm): 9.20 (br, s, amidine), 8.05 (s, 4H, Pz), 7.91 (d, *J* = 8.6 Hz, 8H, Ar), 7.64 (d, *J* = 8.3 Hz, 8H, Ar), 4.98 (t, 4H, *J = 5.5 Hz*, OH), 4.30 (t, 8H, CH_2_), 3.62 (m, 8H, CH_2_), 2.48 (s, 12H, CH_3_); ^13^C-NMR (DMSO-*d*_6_) δ (ppm): 165.2 (amidine), 151.54 (Pz), 149.7 (Ar), 138.31 (Pz), 132.80 (Pz), 129.5 (Ar), 122.2 (Ar), 118.3 (Ar), 59.58 (CH_2_), 48.12 (CH_2_), 14.12 (CH_3_); IR (KBr, cm^−1^): 3353 (C=NH imine/OH alcohol), 3143 (NH_3_^+^), 2925 (CH_2_/ CH_3_), 1680 (NCN/C=N imine), 1608 (triazene), 1572 (CN imidazole ring), 1510 (NH_3_^+^), 1485 (CH_2_/CH_3_), 1440 (C-O alcohol), 1258 (triazene), 1174 (triazene), 1075 (C-O alcohol), 579 (Au-N); MS (ES, HR) *m/z* (M^+^) calcd. for C_52_H_68_Cl_4_N_26_O_12_Au_2_ 1782.3595, found 1782.3600; Anal. calcd. for C_52_H_64_N_26_O_12_Au_2_·4HCl·2H_2_O: C, 34.30; H, 3.99; N, 20.00; found: C, 34.26; H, 3.97 N, 19.91.

#### 4.3.2. Preparation of [Pd_2_(2-(1-methyl-5-nitroimidazol-2-yl)ethanol)_4_(berenil)_2_]·4HCl·2H_2_O (**PdMet-1**)

K_2_PdCl_4_ (1.05 mmol) was dissolved in deionized water (30 mL). Then, 2-(1-methyl-5-nitroimidazol-2-yl)ethanol (2.00 mmol) was added to the reaction mixture and gradual dissolution of the substrate was observed. Stirring was continued for 72 h and the newly formed precipitate was then collected by filtration. This compound (Pd(2-(1-methyl-5-nitroimidazol-2-yl)ethanol)_2_Cl_2_]) was filtered, then washed with deionized water (20 mL) and dried under vacuum. Pd(2-(1-methyl-5-nitroimidazol-2-yl)ethanol)_2_Cl_2_ (0.81 mmol) was suspended in an aqueous solution of silver nitrate (AgNO_3_, 5 mL, 1.62 mmol). The reaction mixture was stirred for 48 h at room temperature in the dark. The AgCl precipitate was filtered off. Berenil (0.81 mmol) and a solution of 10% NaCl (5 mL) were added to the filtrate and stirred for 24 h until a precipitate of the final palladium complex formed. Afterwards, the product was filtered off and washed with a small amount of diluted HCl, deionized water, methanol, and ethyl ether, and dried under vacuum. Yield: 47.7% (309.6 mg); brick-red powder; mp 216–218 °C; ^1^H-NMR (DMSO-*d*_6_) δ (ppm): 9.24 (br, s, amidine), 8.03 (s, 4H, Pz), 7.90 (d, *J* = 8.6 Hz, 8H, Ar), 7.66 (d, *J* = 8.3 Hz, 8H, Ar), 5.02 (t, 4H, *J = 5.5 Hz*, OH), 4.34 (t, 8H, CH_2_), 3.66 (m, 8H, CH_2_), 2.44 (s, 12H, CH_3_); ^13^C-NMR (DMSO-*d*_6_) δ (ppm): 165.2 (amidine), 152.04 (Pz), 149.5 (Ar), 138.51 (Pz), 132.71 (Pz), 129.5 (Ar), 122.0 (Ar), 118.0 (Ar), 59.74 (CH_2_), 48.12 (CH_2_), 14.12 (CH_3_); IR (KBr, cm^−1^): 3381 (C=NH imine/OH alcohol), 3143 (NH_3_^+^), 2925 (CH_2_/ CH_3_), 1687 (NCN/C=N imine), 1606 (triazene), 1572 (CN imidazole ring), 1514 (NH_3_^+^), 1482 (CH_2_/CH_3_), 1440 (C-O alcohol), 1257 (triazene), 1173 (triazene), 1075 (C-O alcohol), 835 (Ar-NO_2_), 518 (Pd-N); MS (ES, HR) *m/z* (M^+^) calcd. for C_52_H_68_Cl_4_N_26_O_12_Pd_2_ 1600.2334, found 1600.2340; Anal. calcd. for C_52_H_64_N_26_O_12_Pd_2_·4HCl·2H_2_O: C, 38.08; H, 4.43; N, 22.21; found: C, 38.06; H, 4.40 N, 22.14.

#### 4.3.3. Preparation of [Pt_2_(2-(1-methyl-5-nitroimidazol-2-yl)ethanol)_4_(berenil)_2_]·4HCl·2H_2_O (**PtMet-1**)

K_2_PtCl_4_ (0.72 mmol) was dissolved in deionized water (30 mL). KI (7.2 mmol) was added and the reaction mixture was stirred for 30 min. Then, 2-(1-methyl-5-nitroimidazol-2-yl)ethanol (2.00 mmol) was added to the reaction mixture and gradual dissolution of the substrate was observed. Stirring was continued for 24 h and the newly formed precipitate was then collected by filtration. This compound [Pt(2-(1-methyl-5-nitroimidazol-2-yl)ethanol)_2_I_2_] was filtered, then washed with deionized water (20 mL) and dried under vacuum. Pt(2-(1-methyl-5-nitroimidazol-2-yl)ethanol)_2_I_2_ (0.57 mmol) was suspended in an aqueous solution of silver nitrate (AgNO_3_, 5 mL, 1.14 mmol). The reaction mixture was stirred for 24 h at room temperature in the dark. The AgI precipitate was filtered off. Berenil (0.57 mmol) and a solution of 10% NaCl (5 mL) were added to the filtrate and stirred for 24 h until a precipitate of the final platinum complex formed. Afterwards, the product was filtered off and washed with a small amount of diluted HCl, deionized water, methanol, and ethyl ether, and dried under vacuum. Yield: 76.5% (389.1 mg); yellow powder; mp 184–186 °C; ^1^H-NMR (DMSO-*d*_6_) δ (ppm): 9.24 (br, s, amidine), 8.03 (s, 4H, Pz), 7.91 (d, *J* = 8.6 Hz, 8H, Ar), 7.68 (d, *J* = 8.3 Hz, 8H, Ar), 5.04 (t, 4H, *J = 5.5 Hz*, OH), 4.36 (t, 8H, CH_2_), 3.69 (m, 8H, CH_2_), 2.48 (s, 12H, CH_3_); ^13^C-NMR (DMSO-*d*_6_) δ (ppm): 165.2 (amidine), 151.84 (Pz), 149.5 (Ar), 138.31 (Pz), 132.84 (Pz), 129.5 (Ar), 122.0 (Ar), 118.0 (Ar), 59.68 (CH_2_), 48.16 (CH_2_), 14.12 (CH_3_); IR (KBr, cm^−1^): 3392 (C=NH imine/OH alcohol), 3143 (NH_3_^+^), 2925 (CH_2_/ CH_3_), 1687 (NCN/C=N imine), 1607 (triazene), 1572 (CN imidazole ring), 1514 (NH_3_^+^), 1485 (CH_2_/CH_3_), 1440 (C-O alcohol), 1259 (triazene), 1175 (triazene), 1075 (C-O alcohol), 835 (Ar-NO_2_), 579 (Pt-N); MS (ES, HR) *m/z* (M^+^) calcd. for C_52_H_68_Cl_4_N_26_O_12_Pt_2_ 1778.3560, found 1778.3556; Anal. calcd. for C_52_H_64_N_26_O_12_Pt_2_·4HCl·2H_2_O: C, 34.37; H, 3.99; N, 20.04; found: C, 34.36; H, 3.97 N, 19.94.

### 4.4. Biological Activity

#### 4.4.1. Cell Lines and Cell Culture

Human breast cancer cell line (MCF-7 and MDA-MB-231) and normal human breast epithelial cell (MCF-10A) were purchased from the American Type Culture Collection (ATCC, Manassas, VA, USA). MCF-7 and MDA-MB-231 cells were cultured in Dulbecco’s Modified Eagle Medium (Gibco, San Diego, CA, USA), MCF-10A cells were cultured in Mammary Epithelial Cell Growth Medium with supplements: BPE, hEGF, Insulin, Hydrocortisone, GA-1000 (Lonza, Basel, Switzerland). All medias were complemented by 10% of fetal bovine serum (FBS) and 1% of antibiotics: penicillin/streptomycin (both Gibco). The cells were maintained in an incubator that provides optimal growth conditions for the cell culture: 5% CO_2_, 37 °C, and humidity in a range of 90−95%. The cells were cultured in 100 mm plates (Sarstedt, Newton, NC, USA). Subsequently after obtaining a subconfluent cell culture, the cells were detached with 0.05% trypsin and 0.02% EDTA phosphate buffered saline without calcium and magnesium (Gibco). Then, utilizing a hemocytometer, the number of cells was quantified and seeded at a density of 5 x 10^5^ cells per well in six-well plates (“Nunc”) in 2 mL of the growth medium (Dulbecco’s Modified Eagle Medium or Mammary Epithelial Cell Growth Medium, respectively). In the present study, cells that obtained 80% of confluence were used.

#### 4.4.2. Cell Viability Assay

The vitality of the cultured cells was investigated using enzymatic reduction of the tetrazolinum dye MTT to formazan. Basically, seed MDA-MB-231, MCF-7, and MCF-10A cells lines at an initial concentration of 1 x 10^5^ cells/well were incubated at 37 °C, 5% CO_2_ for 24 h. Then, the cultured cells were treated with a medium containing various amounts of AuMet-1, PdMet-1, and PtMet-1 (5, 10, 25, 50, and 100 μM) and incubated at 37 °C, 5% CO_2_ for 24 h. After the incubation period, MTT was added (final concentration 0.5 mg/mL) to each well and the plate was incubated at 37 °C, 5% CO_2_ for 4 h. Next, the medium was removed and 200 µL of DMSO was added into each well to dissolve the formazan (0.5% DMSO). The absorbance of the formazan solution was measured at a wavelength of 570 nm (reference – 630 nm) in an Evolution 201 reader (Thermo Scientific, Waltham, MA, USA).

#### 4.4.3. Flow Cytometry Assessment of Annexin V Binding

To determine the mechanism of cell death induced by AuMet-1, PdMet-1, and PtMet-1, the exposure of phosphatidylserine was assessed by annexin V–FITC (phospholipid-binding protein) using the FITC Annexin V Apoptosis Detection Kit II (BD Pharmingen, San Diego, CA, USA), according to the manufacturer’s instructions. A flow cytometer (BD FACSCanto II flow cytometer; BD Biosciences Systems, San Jose, CA, USA) was used to analyze the tested cells (10,000 events measured). Phosphatidylserine was bound with high affinity by Annexin V and thus identification of cells at all stages of apoptosis could be performed using this protein. Propidine iodide (PI) was used to identify late-apoptotic and dead cells because it stains cells with a disrupted cell membrane. The control was cells cultured in a medium without drugs. A positive control (cells incubated with 3% formaldehyde in buffer for 30 min on ice) was used to find the optimal parameter settings. An analysis of the results was performed using FACSDiva software (BD Biosciences Systems).

#### 4.4.4. Analysis of Mitochondrial Membrane Potential

Disruption of the mitochondrial membrane potential (MMP) was measured using the lipophilic cationic fluorochrome (JC-1 MitoScreen kit; BD Biosciences Systems) as described previously [31]. In brief, the unfixed cells were washed, then resuspended in PBS containing 10 mg/mL of JC-1. Next, the cells were incubated for 15 min at room temperature and protected from light, washed and resuspended in PBS. Thus prepared samples were analyzed immediately by flow cytometry BD FACSCanto II, and then the percentage of cells with disturbed MMP was calculated using FACSDiva software (both from BD Biosciences Systems).

#### 4.4.5. Measuring the Number of Autophagosomes and Autolysosomes by Autophagy Assay, Red

To determine the effects of AuMet-1, PdMet-1 and PtMet-1 compounds on the autophagy process of breast cancer cells MCF-7 and MDA-MB-231, an autophagy assay was performed according to the protocol from the manufacturer. The probe was a cell-permeant aliphatic molecule that fluoresces brightly when inserted in the lipid membranes of autophagosomes and autolysosomes (Autophagy Assay, Red kit; ImmunoChemistry Technologies, Bloomington, MN, USA). In short, the unfixed cells were washed and then resuspended in PBS with the added Autophagy Probe, Red solution. Afterwards, the cells were incubated for 30 min at 37 °C in the dark, washed, resuspended in 1× Cellular Assay Bufor and the provided Fixative was added at a volume/volume ratio of 1:5. The samples were measured immediately after preparation by flow cytometry using a BD FACSCanto II system (BD Biosciences Systems). The percentage of cells with autophagy was calculated using FACSDiva software (BD Biosciences Systems).

#### 4.4.6. Antibody Topoisomerase IIα Detection

To check whether the tested compounds induce cell death by damaging DNA directed to topoisomerases, the activity of topoisomerase IIα in breast cancer cells MCF-7 and MDA-MB-231 was measured using antibody Topoisomerase IIα, according to the manufacturer’s instructions. The probe was anti-topoisomerase IIα antibody conjugated to phycoerythrin. In brief, the centrifuged cells were resuspended in 4% formaldehyde and incubated for 15 min at room temperature. The cells were washed by centrifugation with excess PBS. Afterwards, permeabilization was performed by adding ice-cold 90% methanol to the cells and incubating for 60 min in an ice bath. The cells were washed by centrifugation with excess PBS again. Then, they were resuspended in 100 µL of diluted primary antibody, prepared in PBS at a 1:100 dilution, and incubated for 60 min at room temperature in the dark, washed and resuspended in 300 µL PBS. The samples were measured immediately after preparation by flow cytometry using BD FACSCanto II. An analysis of the results was performed using FACSDiva software (both from BD Biosciences Systems).

#### 4.4.7. DNA Fragmentation Assay

In order to identify and examine DNA fragmentation, the method called terminal deoxynucleotidyltransferase-mediated dUTP nick end labeling (TUNEL) was used. In our study we used a commercial assay kit (APO-Direct Kit; BD Pharmingen) in accordance with the manufacturer’s instructions. In brief, the treated cells were fixed with 1% paraformaldehyde in PBS (4 °C, 30min) and then washed in PBS. Afterwards, permeabilization was performed by adding ice-cold 70% ethanol to the cells. Fixed cells were washed twice using the kit wash buffer, then the cells were centrifuged and the supernatant was discarded. In the next step, the cells were resuspended in Labeling Solution DNA and incubated for 1 h at 37 °C with occasional shaking. At the end of the incubation time, rinse buffer was added, then cells were centrifuged, the supernatant was removed and cells were rinsed again. After that, the cell pellet was suspended in the PI/RNase staining buffer and incubated for 30 min at room temperature in the dark. After this time, the cells were immediately analyzed in a flow cytometer BD FACSCanto II, and then the percentage of cells with distinctive DNA strand breaks was calculated using FACSDiva software (both from BD Biosciences Systems).

#### 4.4.8. Zebrafish Handling, Establishment of Xenograft

Breast cancer MCF-7 and MDA-MB-231 cells were labeled before transplantation. The zebrafish embryos were manually dechorionated at 36 h post-fertilization (hpf) and after another 12 h were anesthetized by placement in 0.04 mg/mL ethyl 3-aminobenzoate methanesulfonate tricaine, which is a water soluble, fast-acting anesthetic agent. Zebrafish embryos were then transferred to a thin film of low-melting-point agarose to stabilize the fish in a lateral position. Breast cancer cells were loaded into a borosilicate glass needle pulled by a P-1000 Next Generation Micropipette Puller (Sutter Instrument Company, Novato, CA, USA). A suspension containing about 100–200 cells was injected into the inferior section of the yolk sac in a single injection by using an electronically regulated air-pressure microinjector (Narishige IM-300 Microinjector, Los Angeles, CA, USA). After injection, the zebrafish were washed once with fish water and transferred to a 6-well plate containing 2 mL of fresh fish water. MCF-7 and MDA-MB-231- xenografts (72 hpf) were incubated at 33 °C with PdMet-1 (100 µM) and PtMet-1 (100 µM) for 48 h. Although embryos are normally allowed to develop at 28.5 °C and human cells at 37 °C, a compromise at 33 °C works well.

#### 4.4.9. Microscope Imaging

Living zebrafish embryos were anesthetized by tricaine and embedded in a lateral orientation. The animals were analyzed for cytoplasmic fluorescence intensity. Images of the cells were acquired using an EVOS M5000 Imaging System (Thermo Fisher Scientific Inc., Waltham, MA, USA) with the following filters: GFP (470/22 nm Excitation; 510/42 nm Emission) and Texas Red (585/29 nm Excitation; 624/40 nm Emission). Image analysis was performed using ImageJ v1.51 software (National Institutes of Health, Bethesda, MD, USA).

### 4.5. Statistical Analysis

All numerical data are presented as mean ± standard deviation (SD) from at least 3 independent experiments. Statistical analysis was conducted using the GraphPad Prism 8 software (GraphPad Software, San Diego, CA, USA). Statistical differences in multiple groups were determined by one-way ANOVA followed by Tukey’s test. *p* < 0.05 was considered statistically significant.

## 5. Conclusions

In conclusion, the novel metal complexes may be excellent molecular frameworks that are amenable for optimization for two types of breast cancer cell death: apoptosis and/or autophagy. We discovered that topoisomerase IIα increased activity caused by the tested compounds could potentially lead to DNA fragmentation, resulting in cell death by apoptosis. The outcomes of our study suggest that the novel synthetic transition metal complexes with berenil and nitroimidazole may constitute a new group of anticancer agents for the treatment of breast cancer patients.
